# Increased Cytoplasmic CD138 Expression Is Associated with Aggressive Characteristics in Prostate Cancer and Is an Independent Predictor for Biochemical Recurrence

**DOI:** 10.1155/2020/5845374

**Published:** 2020-10-28

**Authors:** Simon Kind, Martina Kluth, Claudia Hube-Magg, Katharina Möller, Georgia Makrypidi-Fraune, Florian Lutz, Maximilian Lennartz, Sebastian Dwertmann Rico, Thorsten Schlomm, Hans Heinzer, Doris Höflmayer, Sören Weidemann, Ria Uhlig, Hartwig Huland, Markus Graefen, Christian Bernreuther, Maria Christina Tsourlakis, Sarah Minner, David Dum, Andrea Hinsch, Andreas M. Lübke, Ronald Simon, Guido Sauter, Andreas Marx, Adam Polonski

**Affiliations:** ^1^Institute of Pathology, University Medical Center Hamburg-Eppendorf, 20246 Hamburg, Germany; ^2^Department of Urology, Charité-Universitätsmedizin Berlin, Berlin, Germany; ^3^Martini-Clinic, Prostate Cancer Center, University Medical Center Hamburg-Eppendorf, 20246 Hamburg, Germany; ^4^Institute of Pathology, Klinikum Fürth, Fürth, Germany; ^5^General, Visceral and Thoracic Surgery Department and Clinic, University Medical Center Hamburg-Eppendorf, 20246 Hamburg, Germany

## Abstract

Syndecan-1 (CD138) is a transmembrane proteoglycan expressed in various normal and malignant tissues. It is of interest due to a possible prognostic effect in tumors and its role as a target for the antibody-drug conjugate indatuximab ravtansine. Here, we analyzed 17,747 prostate cancers by immunohistochemistry. Membranous and cytoplasmic CD138 staining was separately recorded. In normal prostate glands, CD138 staining was limited to basal cells. In cancers, membranous CD138 positivity was seen in 19.6% and cytoplasmic CD138 staining in 11.2% of 12,851 interpretable cases. A comparison with clinico-pathological features showed that cytoplasmic CD138 staining was more linked to unfavorable tumor features than membranous staining. Cytoplasmic CD138 immunostaining was associated with high tumor stage (*p* < 0.0001), high Gleason grade (*p* < 0.0001), nodal metastases (*p* < 0.0001), positive surgical margin (*p* < 0.0001), and biochemical recurrence (*p* < 0.0001). This also holds true for both V-ets avian erythroblastosis virus E26 oncogene homolog (ERG) fusion positive and ERG fusion negative tumors although the cytoplasmic CD138 expression was markedly more frequent in ERG positive than in ERG negative tumors (*p* < 0.0001). Comparison with 11 previously analyzed chromosomal deletions identified a conspicuous association between cytoplasmic CD138 expression and 8p deletions (*p* < 0.0001) suggesting a possible functional interaction of CD138 with one or several 8p genes. Multivariate analysis revealed the cytoplasmic CD138 expression as an independent prognostic parameter in all cancers and in the ERG positive subgroup. In summary, our study indicates the cytoplasmic CD138 expression as a strong and independent predictor of poor prognosis in prostate cancer. Immunohistochemical measurement of CD138 protein may thus—perhaps in combination with other parameters—become clinically useful in the future.

## 1. Introduction

In 2018 was prostate cancer with more than 350,000 deaths worldwide, the second most common cause for cancer-related death in men [1]. However, many more men got diagnosed with prostate cancer [2]. It is pivotal to properly assess the patient's individual risk of tumor progression to restrict aggressive treatment to avoid treatment-related complications and affection of the quality of life [3–5]. The established prognostic factors (Gleason grade, tumor quantity in biopsies, prostate-specific antigen (PSA) serum values, and clinical stage) are statistically powerful but do not allow clear-cut risk stratification. Therefore, the search for novel biomarkers to improve the assessment of tumor aggressiveness goes on.

CD138 or syndecan-1 (*SDC1*) is a transmembrane heparin sulfate proteoglycan. Its extracellular domain binds heparin sulfates and chondroitin sulfates [6]. CD138 plays an important role for cell-cell and cell-matrix interactions. It is involved in the regulation of cell migration and the organization of the cytoskeleton [6]. In normal tissues, CD138 is expressed on plasma cells and various epithelial cells. Altered expression of CD138 has been described for many neoplasias including cancers of the breast, urinary bladder, pancreas, ovary, and endometrium [6–10]. In several of these tumors, an increased or decreased CD138 expression was found to be linked to unfavorable tumor phenotype and poor patient prognosis [8, 9, 11]. The CD138 expression in cancer is also of clinical interest because specific drugs targeting CD138 are currently being evaluated in clinical trials on multiple myeloma and breast cancer [12–14].

The available data suggest that the increased CD138 expression may also be linked to prostate cancer aggressiveness. Although most of the 15 studies analyzing 18-551 prostate cancers have described associations of the altered CD138 expression with high Gleason grade, high Ki67 labeling index (Ki67LI), early recurrence, tumor-specific survival or elevated expression in hormone-refractory recurrences, and distant metastasis [15–28], there is still no consent on the suitability of CD138 as routine diagnostic marker.

On a genetic level, about 50% of prostate cancer is characterized by gene fusion of the androgen responsive TMPRSS2 serin protease and the ETS-family transcription factor ERG. Other frequent chromosomal alterations include deletions of the PTEN tumor suppressor and other loci including chromosomes 3p, 5q, 8p, 12p, 12q, 13q, 17p, and 18q, most of which are linked to either ERG fusion positive or ERG negative cancers [29]. Here, we took the advantage of a very large preexisting tissue microarray (TMA) containing more than 17,000 prostate cancer specimens to stratify the immunohistochemical CD138 expression for multiple clinical, phenotypic, and genetic parameters.

## 2. Materials and Methods

### 2.1. Patients

The 17,747 patients had radical prostatectomy between 1992 and 2015 (Department of Urology and the Martini Clinic at the University Medical Center Hamburg-Eppendorf). Prostate specimens were analyzed according to a standard procedure for tumor stage, Gleason grade, nodal stage, and resection margin status [30]. In addition to the classical Gleason categories, “quantitative” Gleason grading was performed as described before [31]. Gleason 3+4 and 4+3 cancers were divided into 8 subgroups according to their percentage of Gleason 4 pattern (3 + 4 ≤ 5% Gleason 4, 3+4 6-10%, 3+4 11-20%, 3+4 21-30%, 3+4 31-49%, 4+3 50-60%, 4+3 61-80%, and 4 + 3 > 80% Gleason 4). Two additional groups were defined by the presence of a tertiary Gleason 5 pattern (3+4 Tert.5 and 4+3 Tert.5). Follow-up was available for 14,464 patients (median follow-up 48 months, Table 1). PSA recurrence was defined as the time point when postoperative PSA was at least 0.2 ng/ml and increasing at subsequent measurements. The TMA was produced with one 0.6 mm core taken from a tumor containing tissue block from each patient as described before [32]. The attached molecular database contained data on Ki67LI of 5,492 tumors [33], ERG expression data of 13,089 tumors [34], *ERG* break apart FISH data of 7,225 (expanded from [34]), and androgen receptor (AR) expression data of 7,971 cancers [35] as well as data on the deletion status of 5q21 (*CHD1*) of 8,047 (expanded from [36]), 6q15 (*MAP3K7*) of 6,171 (expanded from [37]), *PTEN* (10q23) of 6,803 (expanded from [38]), 3p13 (*FOXP1*) of 7,201 (expanded from [39]), 13q14 of 7,499 [40], 18q21 of 7,032 [41], 8p21 of 7,001 [42], 12p13 of 6,187 [43], 12q24 of 7,435 [34], 16q24 of 5,493 [44], and 17p13 (*TP53*) of 8,307 cancers [45]. Archived diagnostic leftover tissues for manufacturing of tissue microarrays and their analysis for research purposes as well as patient data analysis have been approved by local laws (HmbKHG §12,1) and by the local ethics committee (Ethics commission Hamburg, WF-049/09). All work has been carried out in compliance with the Helsinki Declaration.

### 2.2. Immunohistochemistry

Freshly cut TMA sections were stained in a single experiment. Slides were deparaffinized and exposed to antigen retrieval for 5 minutes at 121°C in pH 9 Dako Target Retrieval Solution buffer. Primary antibody specific for total Syndecan-1 (mouse monoclonal antibody, clone JASY1, OncoDianova, dilution 1 : 200) was applied at 37°C for 60 minutes. Bound antibody was visualized with the EnVision Kit (Dako, Glostrup, Denmark). Membranous and cytoplasmic CD138 staining of variable intensity was seen in prostate cancer cells whereas no stromal CD138 staining was observed. Therefore, the percentage of positive cells and the staining intensity were separately evaluated for membranous and cytoplasmic staining. Intensity was quantitated as 0 (negative), 1+ (weak), 2+ (moderate), and 3+ (strong). The results of both membranous and cytoplasmic CD138 staining were then further categorized in 4 groups for statistical analyses. Tumors without any staining were considered as negative. Tumors with 1+ staining intensity in ≤70% of cells and 2+ intensity in ≤30% of cells were considered weakly positive. Tumors with 1+ staining intensity in >70% of cells, 2+ intensity in 31% to 70%, or 3+ intensity in ≤30% were considered moderately positive. Tumors with 2+ intensity in >70% or 3+ intensity in >30% of cells were considered strongly positive.

### 2.3. Statistics

The JMP® 10.0.2 software (SAS Institute Inc., NC, USA) was used. Contingency tables and the chi^2^ test were done to look for associations. Kaplan-Meier plots were tested with the log-rank test for differences between groups. Cox proportional hazards regression analysis was performed to test the statistical independence and significance between pathological, molecular, and clinical variables. When indicated, Bonferroni correction was applied for multiple testing.

## 3. Results

### 3.1. CD138 Expression in Normal and Cancerous Prostate Tissues

**Figure 1:**
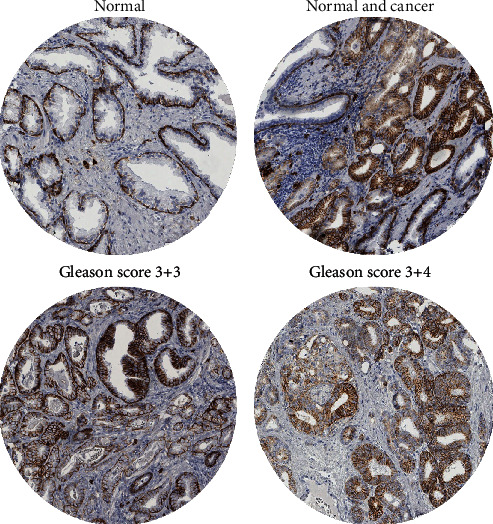
Examples of membranous and cytoplasmic CD138 immunostaining.

**Figure 2:**
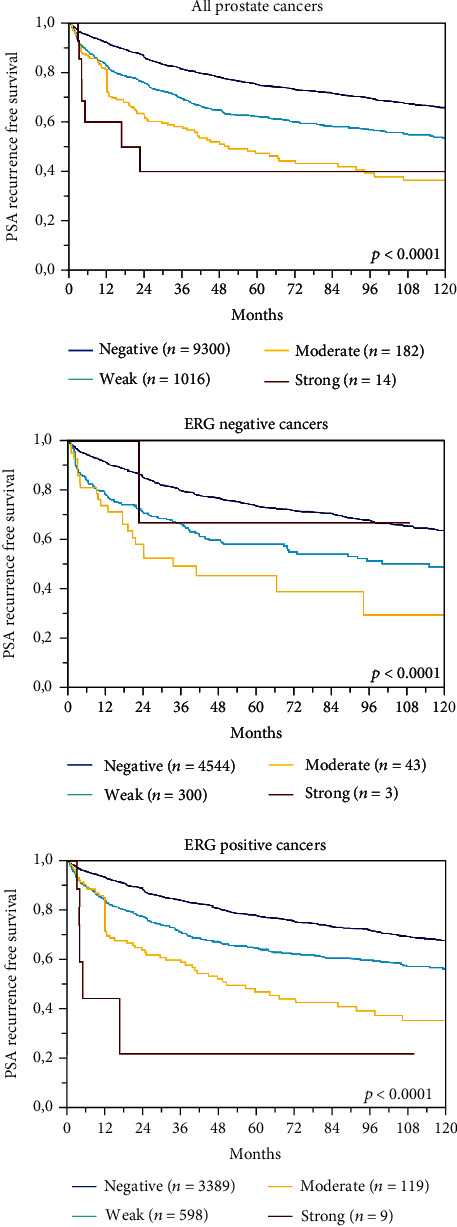
Cytoplasmatic CD138 immunostaining and biochemical recurrence.

**Figure 3:**
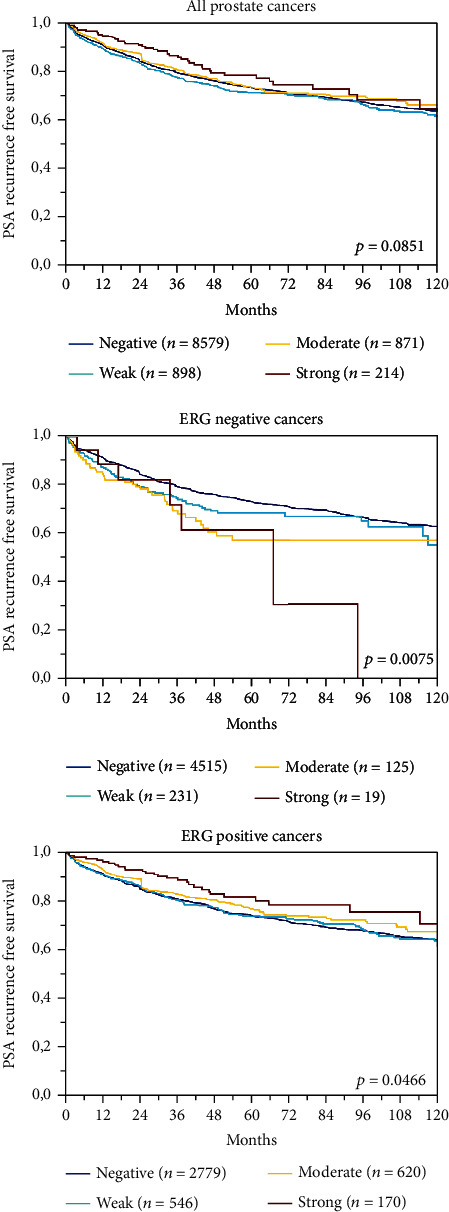
Membranous CD138 immunostaining and biochemical recurrence.

A total of 12,851 (72%) tumor samples were interpretable in our TMA analysis. Noninformative cases (*n* = 4,896; 28%) were caused by lack of tissue or unequivocal tumor cells in the corresponding tumor spot. In normal prostate tissue, basal cells showed a reproducible positive CD138 expression while no staining was detected in glandular cells. In prostate cancer, membranous and/or cytoplasmic CD138 staining was sometimes seen. Cytoplasmic staining appeared as diffuse staining of the entire cytosol, typically in addition to membrane staining. Membranous CD138 expression was seen in 19.6% of cancers from which 9.0% showed weak, 8.3% with moderate, and 2.2% with strong staining. Cytoplasmic CD138 staining was found in 11.2% of 12,851 cancers and was considered weak in 9.5%, moderate in 1.6%, and strong in 0.1%. Examples of CD138 immunostainings are shown in Figure 1. A comparison with tumor phenotype revealed that in particular cytoplasmic CD138 staining was linked to unfavorable tumor features (Table 2) and PSA recurrence (Figure 2). Presence of membranous staining was also associated with unfavorable tumor phenotype and early PSA recurrence, but these relationships were less striking (Table 3; Figure 3). Membranous and cytoplasmic CD138 staining was related. The frequency of cytoplasmic staining increased gradually from 6.1% in cancers without membranous staining to 51.2% in cancers with strong CD138 positivity (*p* < 0.0001).

### 3.2. *TMPRSS2:ERG* Fusion Status and CD138 Expression

**Figure 4:**
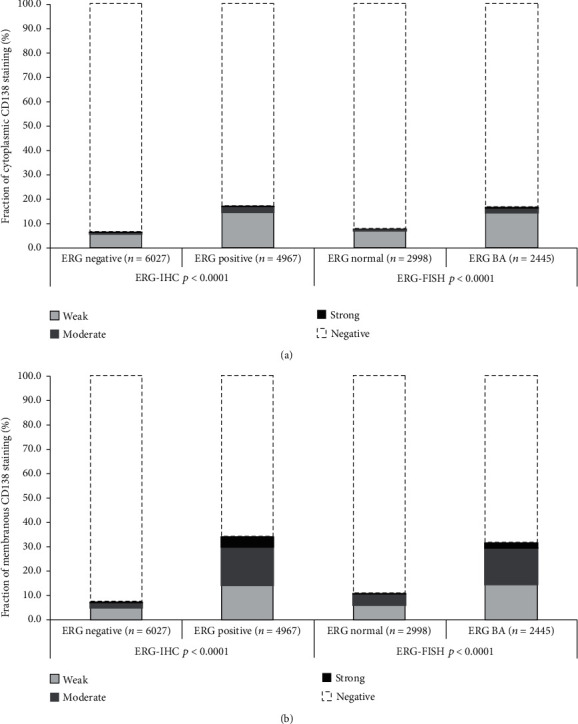
Cytoplasmic (a) and membranous (b) CD138 immunostaining and *TMPRSS2:ERG.*

Data on *TMPRSS2:ERG* fusion status obtained by FISH were available from 5,443 and by IHC from 10,994 tumors with evaluable CD138 immunostaining. Across 4,795 cases, we found a concordance of 91.6% between FISH and IHC analyzed data. Both, membranous and cytoplasmic CD138 immunopositivity, were more than 2 times more frequent in ERG positive than in ERG negative cancers (*p* < 0.0001; Figure 4). Given this considerable difference associations between the CD138 expression, tumor phenotype and PSA recurrence were separately analyzed in ERG positive and negative cancers. All these analyses revealed similar results to the entire patient cohort for both ERG positive and ERG negative cancers (Figures 2 and 3 and Supplementary Table 1 and 2).

### 3.3. CD138 Expression, Key Genomic Deletions, and Tumor Cell Proliferation

**Figure 5:**
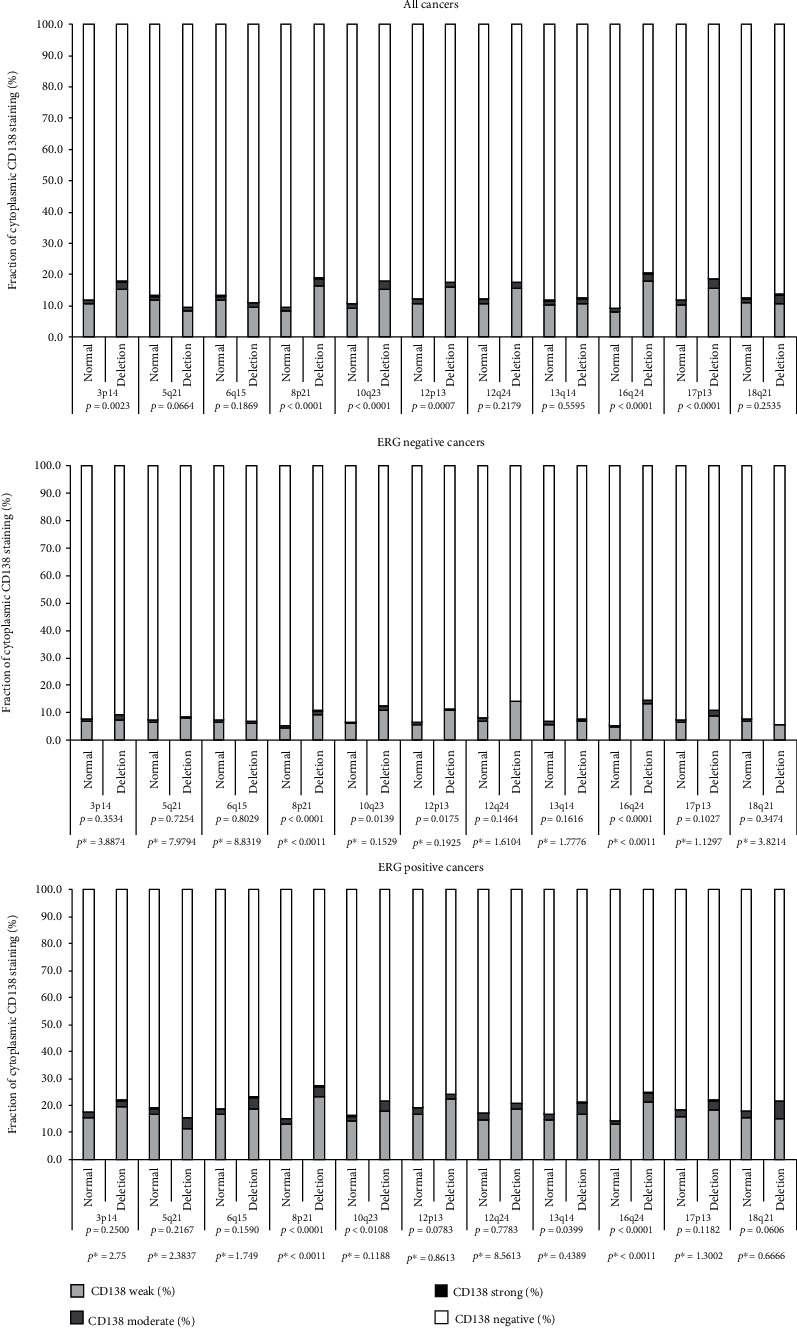
Cytoplasmic CD138 immunostaining and common deletions. *p*^∗^ Bonferroni corrected *p* values.

**Figure 6:**
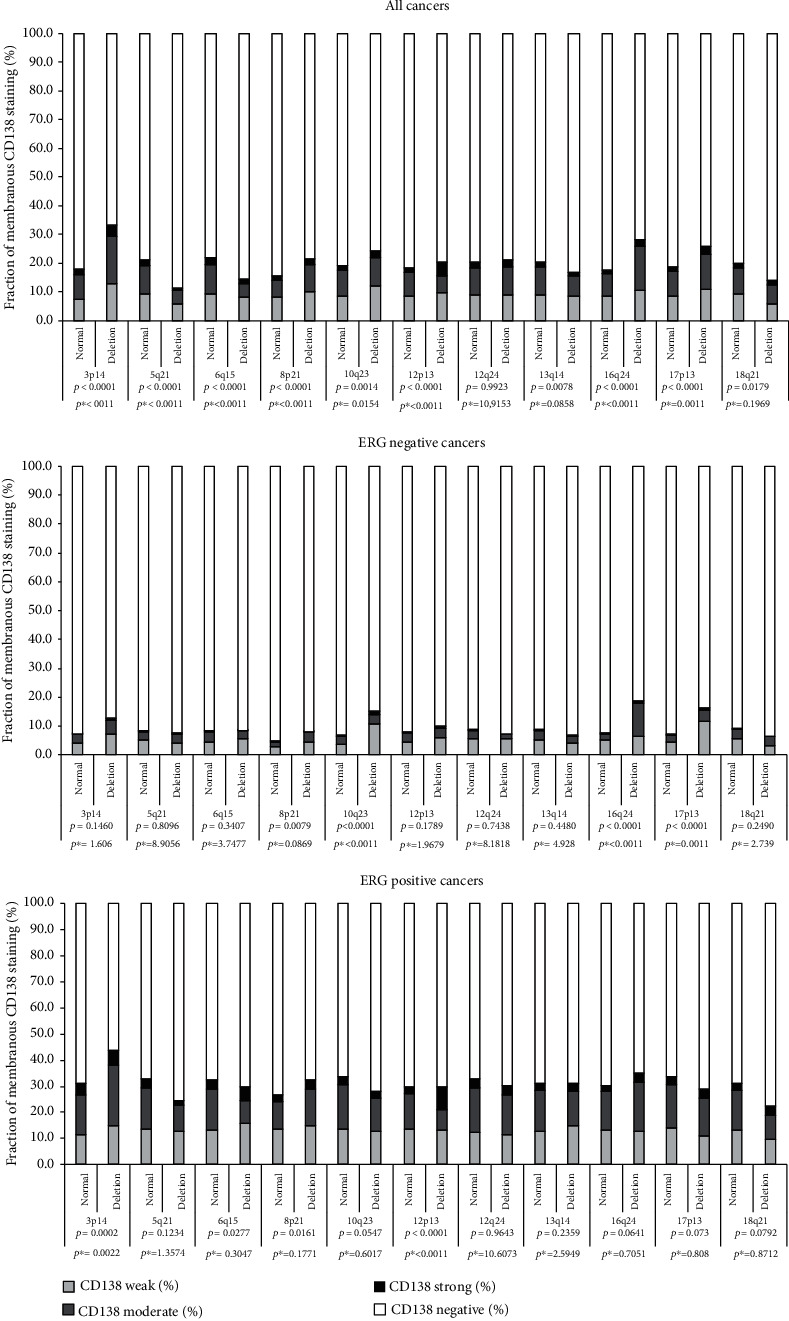
Membranous CD138 immunostaining and common deletions. *p*^∗^ Bonferroni corrected *p* values.

The relationship between CD138 expression and 11 of the most frequent genomic deletions (*PTEN*, 3p13, 5q21, 6q15, 13q14, 18q21, 8p21, 12p13, 12q24, 16q24, 17p13) was not only analyzed for the entire tumor cohort but also for the subgroups with identical ERG status (Figures 5 and 6). The analysis revealed for membranous CD138 staining significant associations with 10 deletions in all cancers, 4 deletions in ERG positive cancers, and 4 deletions in ERG negative cancers. For the cytoplasmic CD138 expression, significant associations were seen with 6 deletions in all cancers, 4 deletions in ERG positive cancers, and 6 deletions in ERG negative cancers. Most statistically significant associations were not striking. It was conspicuous, however, that 8p deletions were strongly linked to membranous and cytoplasmic CD138 positivity in both ERG positive and negative cancers (*p* < 0.0001 in 3 of 4 analyses). Further subset analyses revealed that this association was strongest in low grade cancers (Gleason ≤3+4, *p* < 0.0001) but also retained in high grade cancers (Gleason ≤4+3, *p* = 0.0252). The cytoplasmic and membranous CD138 expression levels were only marginally related to tumor cell proliferation, determined by Ki67LI. Ki67LI was 2.72 ± 0.04 in 5,050 tumors with negative, 2.87 ± 0.11 in 588 tumors with weak, was 2.93 ± 0.28 in 89 tumors with moderate, and was 8.17 ± 1.06 in 6 tumors with strong cytoplasmic CD138 staining (*p* < 0.0001; *p* = 0.0012 after Bonferroni correction). Ki67LI was 2.7 ± 0.04 in 4,688 tumors with negative, 2.86 ± 0.12 in 437 tumors with weak, was 3.1 ± 0.12 in 484 tumors with moderate, and was 2.39 ± 0.23 in 124 tumors with strong membranous CD138 staining (*p* = 0.0039; data not shown).

### 3.4. Multivariate Analysis

To assess whether the prognostic effect of CD138 immunostaining in prostate cancer depended on already established parameters, four different multivariate analyses were performed as previously described [46, 47]: scenario 1 analyzed the postoperative parameters (pT, pN, surgical margin status, preoperative PSA value, Gleason grade, and CD138); in scenario 2, pN was excluded, because the indication and extent of lymph node surgery is not standardized for prostate cancer; scenario 3 included mainly preoperative parameters (clinical tumor stage (cT), preoperative PSA level, prostatectomy Gleason grade, and CD138); and in scenario 4, the Gleason grade on the prostatectomy specimen was replaced by the preoperative biopsy Gleason grade which may be more impacted by sampling errors and subsequent undergrading in more than one third of cases [48]. In all scenarios, the cytoplasmic CD138 expression provided independent prognostic information (*p* < 0.0001 each; Table 4). This association held true in the subset of ERG positive cancers (*p* ≤ 0.02; Table 4). A separate analysis of cancers with identical traditional and quantitative Gleason grade showed a prognostic effect of the CD138 expression in Gleason 3+4 and 4+3 carcinomas (Supplementary Figure 1).

## 4. Discussion

The results of this study show that cytoplasmic CD138 immunostaining is independently linked to unfavorable tumor features and poor patient outcome in prostate cancer.

Under the experimental conditions selected for this study, benign prostate glands completely lacked CD138 staining in epithelial luminal cells while there was a strong expression in gland surrounding basal cells. A comparable pattern has previously been described by Kiviniemi et al. who found a strong CD138 staining in basal cells and only a weak staining of the basolateral cell membrane of apical cells [19]. Also, Zellweger et al. reported the CD138 expression to only occur in the basal cell layer in benign prostate glands [11]. Pathologists often use IHC to verify cancer diagnosis in prostate tissue by ensuring the absence of basal cells in prostate cancer glands by basal-cell-specific antibodies HMWCK or p63. A possible utility of CD138 immunostaining as a prostate basal cell marker applicable for cancer detection was thus earlier suggested [18].

That CD138 immunostaining was seen in glandular cells of a fraction of prostate cancers argues for overexpression of CD138 to occur during prostate cancer development. In the present study, a membranous CD138 immunostaining was seen in 19.6%, and a cytoplasmic positivity was observed in 11.2% of prostate cancers. These percentages are lower than those from earlier studies, only some of which also reported both cytoplasmic and membranous staining patterns. A membranous CD138 immunostaining was earlier described in 26% of 196 localized cancers [15], 83% of 5 metastatic cancers [15], 35% of 42 hormone-refractory cancers [15], 19% of 103 [25], and 36% of 60 cancers [20]. A cytoplasmic CD138 positivity was earlier recorded in 59% of 103 cancers [25], 64% of 60 cancers [20], 50% of 5 metastatic cancers [15], 32% of 42 hormone-refractory cancers [15], and 47% of 196 localized cancers [15]. Studies not distinguishing between membranous and cytoplasmic staining described CD138 positivity in 37% of 501 [11], 37% of 232 [22], 96% of 54 cancers [49], 38% of 32 Gleason sum 6 cancers [16], 32% of 44 Gleason sum 7 cancers [16], 38% of 99 hormone-refractory cancers [26], 3% of 151 localized cancers [26], and 42% of 12 cancers [19].

The striking association of the CD138 overexpression with patient outcome is the most relevant finding of this study. Associations of the high CD138 expression with unfavorable tumor phenotype, such as early recurrence, high Gleason grade, and poor patient outcome, had already been suggested by other studies investigating 18-551 prostate cancers by IHC [11, 15, 16, 19–22, 24–26]. Moreover, a high serum level of shed soluble CD138 protein was found to be linked to poor prognosis in another cohort of 150 prostate cancer patients [25]. The large number of cases involved in our study enabled us to differentiate the prognostic impact of membranous and cytoplasmic staining. The predominant prognostic effect of cytoplasmic staining fits with one earlier report by Contreras et al. [17] and Ledezma et al. [20] also describing a prognostic effect of cytoplasmic staining. That cytoplasmic CD138 represents an ominous feature in neoplasia is further supported by a recent own study on 1,535 breast cancers where cytoplasmic CD138 was linked to poor prognosis while membranous CD138 staining predicted a favorable disease outcome [50]. In the same study, a favorable prognostic impact of stromal CD138 staining was seen in breast cancer [50]. Stromal cell CD138 immunostaining was earlier reported to occur in prostate cancer [23, 24], but this was not observed in our prostate cancer analysis.

CD138 is a membrane protein with largely unknown cytoplasmic functions. A possible explanation for the cytoplasmic CD138 protein accumulation, it must be considered that “membranous CD138 negativity” by IHC does not equal complete lack of CD138 protein in cell membranes. Immunohistochemical CD138 negativity of membranes only means that the CD138 protein expression level is below the detection threshold of our experimental procedure. The cytoplasmic accumulation of CD138 could potentially be explained by a protein defect or a functional mechanism preventing incorporation of CD138 into the membrane. It has also been suggested that increased CD138 shedding from cell membrane may induce the high CD138 expression and cytoplasmic CD138 accumulation in the cell, an aberrant condition that can develop during cellular dedifferentiation [50]. It appears possible that the extracellular enzyme heparanase (HPSE) plays a role in CD138 shedding by inducing proteolytic cleavage with increasing metastatic potential as result. Heparanase, which also contributes to de novo synthesis of CD138, has been shown to be overexpressed in several tumors, also in prostate cancer (summarized in [20]). The significant association between membranous and cytoplasmic CD138 expression found in our cancers would equally support the concepts of inefficient membrane integration or shedding of CD138.

The highly annotated TMA permitted us to investigate the correlation of CD138 alterations with other parameters of interest. In the present study, we selected *TMPRSS2:ERG* fusion as it represents the most common genomic alteration in prostate cancer, 11 chromosomal deletions because deletions are the next most prevalent genomic alterations in prostate cancer and tumor cell proliferation. *TMPRSS2:ERG* fusions occur in almost half of all prostate cancers and induce overexpression of the transcription factor ERG. ERG expression lacks prognostic relevance but modifies the expression of more than 1,600 genes in affected prostate epithelial cells [51]. Therefore, many proteins are upregulated or downregulated in ERG positive cancer cells in comparison to ERG negative cancer cells or normal tissues [51]. In our study, both expression and the cytoplasmic shift of CD138 are associated with the ERG expression. Shedding of CD138 from the surface is regulated at multiple levels. For examples, by agents that mediate cellular response to stress accelerated shedding or physiological agents that are involved in activation of distinct intracellular signaling pathways [52]. CD138-induced cancer-related pathways are, for example, ERK MAP and JNK MAP Kinase pathways as well as TGF-*ß* signaling [53]. That ERG expression is induced by ERK MAP Kinase pathway [54, 55] might, thus, explain the predominance of the CD138 expression in ERG positive cancers.

As most chromosomal deletions are either linked to ERG positive or negative cancer [36–45], it was expected that several deletions are statistically linked to the CD138 expression which is also ERG related. That subgroup analyses of ERG positive and negative cancers highlighted associations of the CD138 expression and 8p deletions but otherwise showed few strong associations argues against a role of the aberrant CD138 expression for development of a genomic instability that results in double strand breaks. That the link between 8p deletion and CD138 overexpression was also retained in Gleason ≥4+3 cancers argues against a mere coincidence of these alterations in advanced tumors but raises the possibility of a functional interaction of the aberrant CD138 expression and one or several genes on 8p. However, little is currently known about which genes on 8p are most critical for prostate cancer. That no strong link was seen between CD138 expression and tumor cell proliferation argues against a relevant role of CD138 dependent processes for cell cycle control. Zellweger et al., however, earlier reported a correlation between high CD138 expression and high (>10%) Ki67LI, in a cohort of 501 prostate cancers [11].

The strong independent prognostic role of cytoplasmic CD138 immunostaining found in this study raises the possibility that CD138 measurement could provide clinically relevant prognostic information for patients. As prostate cancer is the only cancer where “active surveillance” represents an option, prognosis assessment is critical in this disease. The Gleason score is regarded the most powerful prognostic parameter which is preoperatively available, but it suffers from an interobserver variability reaching up to 40%, even between expert pathologists [56]. Future prognostic biomarkers for prostate cancer should not only be independent of currently established factors but better reproducible and thus more reliable. Given the pivotal role of the intracellular distribution of CD138, the protein represents a prognostic biomarker that probably can only be assessed by IHC. IHC is, however, notorious for reproducibility issues [57]. The advent of multiplex IHC enabling the simultaneous application of multiple antibodies also including internal controls and a computerized quantification of individual proteins may assist in making this possible.

## 5. Conclusions

The successful immunohistochemical analysis of 12,851 prostate cancers demonstrated a strong and largely independent association of cytoplasmic CD138 immunostaining with poor patient prognosis in prostate cancer. We consider it likely that a clinically applicable prognostic assay will consist of multiple different parameters to be measured simultaneously. The CD138 protein holds potential for becoming a component of such as a future prognostic tool.

## Figures and Tables

**Table 1:** Study cohort.

	No. of patients (%)	
	Study cohort on TMA (*n* = 17,747)	Biochemical relapse among categories
Follow-up (mo)		
*n*	14464 (81.5%)	3612 (25%)
Mean	56.3	—
Median	48	—
Age (y)		
≤50	433 (2.4%)	66 (15.2%)
51-59	4341 (24.5%)	839 (19.3%)
60-69	9977 (56.4%)	2073 (20.8%)
≥70	2936 (16.6%)	634 (21.6%)
Pretreatment PSA (ng/ml)		
<4	2225 (12.6%)	313 (14.1%)
4-10	10520 (59.6%)	1696 (16.1%)
10-20	3662 (20.8%)	1043 (28.5%)
>20	1231 (7%)	545 (44.3%)
pT stage (AJCC 2002)		
pT2	11518 (65.2%)	1212 (10.5%)
pT3a	3842 (21.7%)	1121 (29.2%)
pT3b	2233 (12.6%)	1213 (54.3%)
pT4	85 (0.5%)	63 (74.1%)
Gleason grade		
≤3+3	3570 (20.3%)	264 (7.4%)
3+4	9336 (53%)	1436 (15.4%)
3+4 Tert.5	798 (4.5%)	165 (20.7%)
4+3	1733 (9.8%)	683 (39.4%)
4+3 Tert.5	1187 (6.7%)	487 (41%)
≥4+4	999 (5.7%)	531 (53.2%)
pN stage		
pN0	10636 (89.4%)	2243 (21.1%)
pN+	1255 (10.6%)	700 (55.8%)
Surgical margin		
Negative	14297 (80.8%)	2307 (16.1%)
Positive	3388 (19.2%)	1304 (38.5%)

Percent in the column “Study cohort on TMA” refers to the fraction of samples across each category. Percent in column “Biochemical relapse among categories” refers to the fraction of samples with biochemical relapse within each parameter in the different categories. Numbers do not always add up to 17,747 in the different categories because of cases with missing data. AJCC: American Joint Committee on Cancer.

**Table 2:** Cytoplasmic CD138 staining and prostate cancer phenotype.

Parameter	*n* evaluable	Negative (%)	Weak (%)	Moderate (%)	Strong (%)	*p* value
All cancers	12851	88.8	9.5	1.6	0.1	
Tumor stage						<0.0001
pT2	8124	92.4	6.6	0.9	0.0	
pT3a	2967	85.0	12.4	2.5	0.2	
pT3b-pT4	1709	78.1	18.1	3.3	0.5	
Gleason grade						<0.0001
≤3+3	2176	92.1	6.9	0.9	0.0	
3+4	7016	90.9	7.9	1.1	0.0	
3+4 Tert.5	632	87.7	10.6	1.7	0.0	
4+3	1269	82.6	13.7	3.5	0.2	
4+3 Tert.5	952	83.6	14.3	1.9	0.2	
≥4+4	685	76.8	18.5	3.5	1.2	
Quantitative Gleason grade						
≤3+3	2176	92.1	6.9	0.9	0.0	<0.0001
3 + 4 ≤ 5%	1683	93.5	5.6	0.8	0.1	
3+4 6-10%	1786	92.1	7.2	0.6	0.1	
3+4 11-20%	1577	91.6	7.4	1.0	0.0	
3+4 21-30%	806	88.2	9.9	1.9	0.0	
3+4 31-49%	614	87.9	9.3	2.8	0.0	
3+4 Tert.5	632	87.7	10.6	1.7	0.0	
4+3 50-60%	522	87.0	12.1	1.0	0.0	
4+3 Tert.5	952	83.6	14.3	1.9	0.2	
4+3 61-<100%	564	80.0	13.8	6.0	0.2	
≥4+4	600	77.5	18.0	3.5	1.0	
Lymph node metastasis						<0.0001
N0	7842	88.6	9.7	1.6	0.2	
N+	935	79.0	18.2	2.5	0.3	
Preoperative PSA level (ng/ml)						<0.0001
<4	1457	91.1	7.1	1.6	0.3	
4-10	7580	90.3	8.3	1.3	0.1	
10-20	2761	86.4	11.8	1.6	0.1	
>20	976	80.7	15.6	3.4	0.3	
Surgical margin						<0.0001
Negative	10114	89.7	8.9	1.3	0.1	
Positive	2691	85.3	11.6	2.7	0.4	

**Table 3:** Membranous CD138 staining and prostate cancer phenotype.

Parameter	*n* evaluable	Negative (%)	Weak (%)	Moderate (%)	Strong (%)	*p* value
All cancers	12851	80.4	9.0	8.3	2.2	
Tumor stage						<0.0001
pT2	8124	81.5	8.1	8.2	2.3	
pT3a	2967	78.9	10.2	8.7	2.2	
pT3b-pT4	1709	78.4	11.7	8.4	1.5	
Gleason grade						<0.0001
≤3+3	2176	84.5	7.4	6.7	1.5	
3+4	7016	79.0	9.2	9.2	2.5	
3+4 Tert.5	632	82.6	8.2	7.9	1.3	
4+3	1269	80.5	9.5	7.9	2.1	
4+3 Tert.5	952	80.1	10.1	7.9	1.9	
≥4+4	685	82.0	10.4	6.0	1.6	
Quantitative Gleason grade						
≤3+3	2176	84.5	7.4	6.7	1.5	<0.0001
3 + 4 ≤ 5%	1683	80.7	8.2	8.3	2.8	
3+4 6-10%	1786	79.3	9.1	8.8	2.8	
3+4 11-20%	1577	78.3	10.4	9.6	1.7	
3+4 21-30%	806	77.4	7.6	10.9	4.1	
3+4 31-49%	614	79.5	8.1	10.3	2.1	
3+4 Tert.5	632	82.6	8.2	7.9	1.3	
4+3 50-60%	522	80.5	9.8	8.2	1.5	
4+3 Tert.5	952	80.1	10.1	7.9	1.9	
4+3 61-<100%	564	82.6	7.8	7.1	2.5	
≥4+4	600	81.7	10.3	6.3	1.7	
Lymph node metastasis						0.2053
N0	7842	80.1	9.5	8.3	2.1	
N+	935	79.1	11.2	8.2	1.4	
Preoperative PSA level (ng/ml)						<0.0001
<4	1457	79.1	9.6	9.3	2.0	
4-10	7580	79.6	9.1	8.9	2.5	
10-20	2761	82.2	8.6	7.4	1.8	
>20	976	84.7	9.0	5.2	1.0	
Surgical margin						0.3745
Negative	10114	80.2	9.1	8.4	2.3	
Positive	2691	81.4	8.7	8.1	1.8	

**Table 4:** Multivariate Cox regression analysis including established prognostic parameters and cytoplasmic CD138 staining in all prostate cancers, in ERG negative and ERG positive subset.

Tumor subset	Scenario	*n* analyzable	*p* value							
			Preoperative PSA level	pT stage	cT stage	Gleason grade prostatectomy	Gleason grade biopsy	pN stage	*R* stage	CD138 cytoplasmic expression
All cancers	1	6885	<0.0001	<0.0001	—	<0.0001	—	<0.0001	<0.0001	<0.0001
	2	10372	<0.0001	<0.0001	—	<0.0001	—	—	<0.0001	<0.0001
	3	10214	<0.0001	—	<0.0001	<0.0001	—	—	—	<0.0001
	4	8746	<0.0001	—	<0.0001	—	<0.0001	—	—	<0.0001
ERG negative cancers	1	3221	<0.0001	<0.0001	—	<0.0001	—	<0.0001	0.0158	0.1018
	2	4823	<0.0001	<0.0001	—	<0.0001	—	—	<0.0001	0.0651
	3	4759	<0.0001	—	<0.0001	<0.0001	—	—	—	0.0024
	4	3918	<0.0001	—	<0.0001	—	<0.0001	—	—	<0.0001
ERG positive cancers	1	2615	<0.0001	<0.0001	—	<0.0001	—	<0.0001	<0.0001	0.0108
	2	4053	<0.0001	<0.0001	—	<0.0001	—	—	<0.0001	<0.0001
	3	3980	<0.0001	—	<0.0001	<0.0001	—	—	—	<0.0001
	4	3447	<0.0001	—	<0.0001	—	<0.0001	—	—	<0.0001

## Data Availability

Data are available from the corresponding author on reasonable request.

## Supplementary Materials

Supplementary Materials Supplementary Figure 1: cytoplasmic CD138 expression and PSA recurrence free survival in subgroups of classical and quantitative Gleason grade. Supplementary Table 1: cytoplasmic CD138 immunostaining and cancer phenotype in ERG subgroups. Supplementary Table 2: membranous CD138 immunostaining and cancer phenotype in ERG subgroups.
