# Supplementary material for: Increased Cytoplasmic CD138 Expression Is Associated with Aggressive Characteristics in Prostate Cancer and Is an Independent Predictor for Biochemical Recurrence
Source: Biomed Res Int. 2020 Oct 28;2020:5845374. doi: 10.1155/2020/5845374 (PMC7641694; doi:10.1155/2020/5845374)
Supplement: Supplementary Materials — Supplementary Figure 1: cytoplasmic CD138 expression and PSA recurrence free survival in subgroups of classical and quantitative Gleason grade. Supplementary Table 1: cytoplasmic CD138 immunostaining and cancer phenotype in ERG subgroups. Supplementary Table 2: membranous CD138 immunostaining and cancer phenotype in ERG subgroups. [file 5845374.f1.docx]

**Supplementary materials**

**Increased cytoplasmic CD138 expression is associated with aggressive characteristics in prostate cancer and is an independent predictor for biochemical recurrence**

Simon Kind^1^, Martina Kluth^1^, Claudia Hube-Magg^1^, Katharina Möller^1^, Georgia Makrypidi-Fraune^1^, Florian Lutz^1^, Maximilian Lennartz^1^, Sebastian Dwertmann Rico ^1^, Thorsten Schlomm^2^, Hans Heinzer^3^, Doris Höflmayer^1^, Sören Weidemann^1^, Ria Uhlig^1^, Hartwig Huland^3^, Markus Graefen^3^, Christian Bernreuther^1^, Maria Christina Tsourlakis^1^, Sarah Minner^1^, David Dum^1^, Andrea Hinsch^1^, Andreas Lübke^1^, Ronald Simon^1*^, Guido Sauter^1^, Andreas Marx^1,4^, Adam Polonski^5^

**Supplementary Figure 1.** Cytoplasmic CD138 expression and PSA recurrence free survival in subgroups of classical and quantitative Gleason grade

**^^**

Supplementary table 1. Cytoplasmic CD138 immunostaining and cancer phenotype in ERG subgroups.

**^^**

Supplementary table 2. Membranous CD138 immunostaining and cancer phenotype in ERG subgroups.

**^^**
